# Suppose You Retired from Business Now?

**Published:** 1921-03

**Authors:** O. F. Lewis


### SUPPOSE YOU RETIRED FROM BUSINESS NOW?

BY O. F. LEWIS

Edward Bok, for thirty years editor of the *Ladies' Home Journal,* has retired from business, while still in full working vigor.

Why?

Just because he wanted to "play," and because he refused (as he said) to "drop in the harness."

"Play" may mean frittering away precious hours in profitless and even unhealthy pastimes, dangerous to self, family and to society.

But to Mr. Bok "play" means cultural diversions, more time for good friends, fine sportsmanship, good health, and for the satisfaction of his stored-up longings and dreams of many years, years when business would not give him the leisure time to play.

We fool ourselves often and are content with talking about business as the game of life—a desperately grim game—and so we go on with it and "drop in the harness."

Mr. Bok is very wise—but he can afford to be. He retires. What of hundreds of thousands of us who can not yet afford to retire?

We can also be wise, within the limitations of business. We can play, in our leisure time, sensibly, profitably. Play is today being preached as never before. It is hailed as a great natural tonic, a medicine. Walter Camp urges one kind of play—regular health exercises. To Sir Edward Grey, fishing and hunting are indispensable.

Field Marshal Haig withdrew in the midst of intolerable pressure on the Western front, from time to time, and played golf on the quiet links of France. We loved Theodore Roosevelt for his sportsman's adventures.

But all these men did something themselves when they played.

They weren't contented simply to sit down and watch a Ruth or a Dempsey, or to follow a Chick Evans about the links, or gaze upon a Tilden. They played actively themselves!

Labor-saving devices, shorter hours and the closed saloon are producing more leisure time in all of our lives. In a city of 125,000 there are in each day a half-million leisure hours.

How will they be spent? For America's good? In what kinds of play—or diversions—or satisfactions of hobbies—or what?

A national organization, called Community Service, with headquarters at 1 Madison Ave., New York, has actually been organized to assist communities all over the country to put into the leisure hours of life abundant chances *for all the people* to enjoy life with play and profit.

Countless communities now almost starve for decent, varied, helpful amusement and the inspiration that comes from the fellowship of play.

Have you read Sinclair Lewis' "Main Street"—the story of a middle-Minnesota community? Strange that play, the most natural thing in the world, should have had to wait so long to begin to come into its own in the life of the sensible, normal man!—*Bertrand Brown, War Camp Community Service.*

